# King's College Hospital

**Published:** 1906-06-02

**Authors:** 


### KING'S COLLEGE HOSPITAL.

The King's College Hospital Festival Dinner was held at the Hôtel Cecil on Monday, Lord Milner presiding. He was supported by Lord Methuen, the Marquess of Winchester, Sir E. C. K. Ollivant, Sir Edward Stern, Sir Robert Douglas, Sir C. Morrison Bell, Sir John Thornycroft, Sir William Bousfield, the Master of the Drapers' Company, and the Mayor of Holborn.

In proposing "Success to King's College Hospital," Lord Milner, who is an old student of King's College, said that whether looked at from the point of view of past achievements or in its two main aspects, the alleviation of human suffering and the skill of medical art and science, or from the point of view of its present efforts to meet the growing requirements of the community, King's College Hospital had a very strong hold upon public sympathy and support. He would not dwell on the time-honoured theme of what King's College Hospital had done in the past or was doing in the present. He was more concerned with the promise of the future. King's College Hospital had engaged in a vast new enterprise, which only the continued sympathy and sustained generosity of the public could bring to a completely successful conclusion. He referred, of course, to the large new hospital which was being erected in South

London. The great stride forward which that represented could only be realised if they bore in mind that while the present hospital cost a sum of £60,000 the new hospital was estimated to cost £300,000; that while the present building occupied about an acre, the new institution would occupy twelve acres; that while the present hospital provided 244 beds, the new institution would provide for no less than 600; and that while the accommodation for out-patients and for pathological research in the present building was exceptionally cramped and inadequate, it was intended that the new institution should have all the arrangements in that respect which would meet the highest scientific requirements of modern progress. Not only was the new hospital to be much larger than the existing one, but it was also to be placed in a position which would enable it to be, even in proportion to its size, vastly more beneficent to the community than the old one. The existing hospital, when started, was in the heart of a very large, dense, and poor population, and although fortunately (because it is a consequence of the progress of the metropolis) that poor population had now left its neighbourhood, and the number of its out-patients was consequently falling off, yet in the new district to which the hospital was about to be transferred there would be an immensely increased number of out-patients. Instead of being in the centre of a district mainly of large buildings, inhabited by a number of prosperous people, the new hospital would be in the heart of a great and growing population, which up to the present time had been singularly deficient in hospital accommodation. Therefore he thought that he was justified in saying that this enterprise in which King's College Hospital was engaged was one of very exceptional magnitude and beneficence. It was a very bold undertaking, and only the most robust faith in the old adage *fortes fortuna juvat* would justify them in having undertaken it. That confidence had so far been remarkably justified. The support which the public had hitherto given had been a very generous one, but in spite of that it was necessary to go on reminding the public of the vast benefits which the hospital was calculated to bestow in order that it might achieve a really unequivocal success. (Applause.)

Lord Methuen, in responding, referred to the success of the bazaar which had recently been held in aid of King's College Hospital at Lincoln's Inn, and thanked most warmly those ladies and gentlemen who had so energetically worked for its success.

The collection at the dinner amounted to £3,382.

